# Hepatoprotective and *in vitro* antioxidant effects of native depolymerised-exopolysaccharides derived from *Termitomyces albuminosus*

**DOI:** 10.1038/s41598-017-04171-0

**Published:** 2017-06-20

**Authors:** Huajie Zhao, Juan Li, Jianjun Zhang, Xiuxiu Wang, Min Liu, Chen Zhang, Le Jia

**Affiliations:** 1 College of Life Science, Shandong Agricultural University, Taian, 271018 PR China; 2 Key Laboratory of Plant Nutrition and Fertilizer, Ministry of Agriculture/Institute of Agricultural Resources and Regional Planning, Chinese Academy of Agricultural Sciences, Beijing, 100081 PR China

## Abstract

In this study, native depolymerised-exopolysaccharides (DEPS) were successfully derived from the exopolysaccharides (EPS) of *Termitomyces albuminosus*, and its hepatoprotective effects against a high-fat emulsion and *in vitro* antioxidant activities were investigated. Based on the results of *in vitro* assays, DEPS showed superior antioxidant activities compared with EPS dose-dependently. According to the *in vivo* assays both EPS and DEPS significantly decreased the lipid levels, improved the enzymatic activities, and reduced lipid peroxidation in both serum and hepatic homogenates. Furthermore, EPS and DEPS attenuated the high-fat emulsion-induced histopathological injury to the liver. Both EPS and DEPS might be used as natural drugs to treat and protect against hyperlipidaemia and liver injury induced by a high-fat emulsion. In addition, based on the results of GC and HPLC analyses, rhamnose and low molecular weight compounds may play an important role in contributing to the antioxidant activities of EPS and DEPS.

## Introduction

Many current epidemic and metabolic syndromes, such as hyperlipidaemia and fatty liver characterized by a group of metabolic risks, are commonly caused by a high-fat diet and a sedentary lifestyle^1^. Hyperlipidaemia, a lipid metabolism disorder deemed to be a major risk factor for fatty liver, hypertension, myocardial infarction, atherosclerosis, stroke and cerebrovascular diseases, clinically presents as aheterogeneous groups of disorders with increased levels of total cholesterol (TC), triglyceride (TG) and low density lipoprotein cholesterol (LDL-C) as well as decreased levels of high-density lipoprotein cholesterol (HDL-C)^2–4^. Oxidative stress has been shown to promote the pathogenic development of hyperlipidaemia and its complications^5^. Furthermore, the generation of oxygen free radicals, which are generally known as reactive oxygen species (ROS) and include hydrogen peroxide, hydroxyl radicals, 2,2-diphenylpicrylhydrazyl (DPPH) and superoxide lipid peroxyl radical, have important roles in the cellular and DNA damage caused by oxidative stress^6, 7^. Hence, antioxidant supplements are very helpful in preventing and treating the damage induced by free radicals, resulting in fewer potential mutations^8^. Moreover, many publications have also showed the hepatoprotective effects of a substance are related to its antioxidant activity^9^. Clinically, hyperlipidaemia has been treated with statins, bile acid sequestrants, ezetimibe fibrates and niacin. However, these synthetic drugs do not treat all aspects of dyslipidaemia and are restricted by their adverse effects. Therefore, an increasing number of researchers are exploiting and manufacturing natural hypolipidaemic substances^4^.

As a well-known symbiotic wild mushroom with termites that is mostly found in Africa and Asia, *Termitomyces albuminosus* has been reported to contain many bioactive components, such as proteins, amino acids, polysaccharides, lipids, saponins, and ergosterol^10, 11^. Fungal polysaccharides, the most abundant substance that includes extracellular, mycelial and intracellular polysaccharides, admittedly exhibited comprehensive biological activities, including hypolipidaemic, antioxidant, hypoglycaemic, and hepatoprotective activities, contributing to the applications of natural products and drugs^12^. Exopolysaccharides (EPS), a high molecular weight biosynthetic polymer, either remains attached to the cell surface or is released into the fermentation liquor. EPS have been shown to possess many beneficial bioactivities, including antioxidant, antitumour, hepatoprotective, blood lipid-lowering, analgesic, anti-inflammatory and anti-ageing properties^13–16^. Furthermore, EPS showed advantages superior to other polysaccharides extracted from the fruiting body and mycelial polysaccharides, including a low cost, ease of purification, a short processing time, and higher yields, indicating that EPS was amenable to large scale industrial production for future applications in nutrition and therapeutics^13^. Based on previous studies by academic researchers, EPS and mycelial polysaccharides from *T*. *albuminosus* possess analgesic, anti-inflammatory and hypolipidaemic properties^14, 17^. However, few reports have been published describing the purification, characterization, and antioxidant and antihyperlipidaemic activities of EPS.

This work was designed to investigate the pharmacological effects of EPS and DEPS (depolymerised-exopolysaccharides) derived from *T*. *albuminosus*, including *in vitro* antioxidant activities as well as hepatoprotective and hypolipidaemic activities in mice with high-fat emulsion-induced hyperlipidaemia.

## Results

### Total polysaccharide analysis

The crude polysaccharide (76.43 g) was obtained from the fermentation broth. After purifying the crude polysaccharide (2 g), the collected pure polysaccharide weighed 1.574 g. Therefore, the polysaccharide yield was 78.70%, indicating that the EPS (60.15 g) was obtained *via* liquid fermentation.

### *In vitro* antioxidant activity

**Figure 1:**
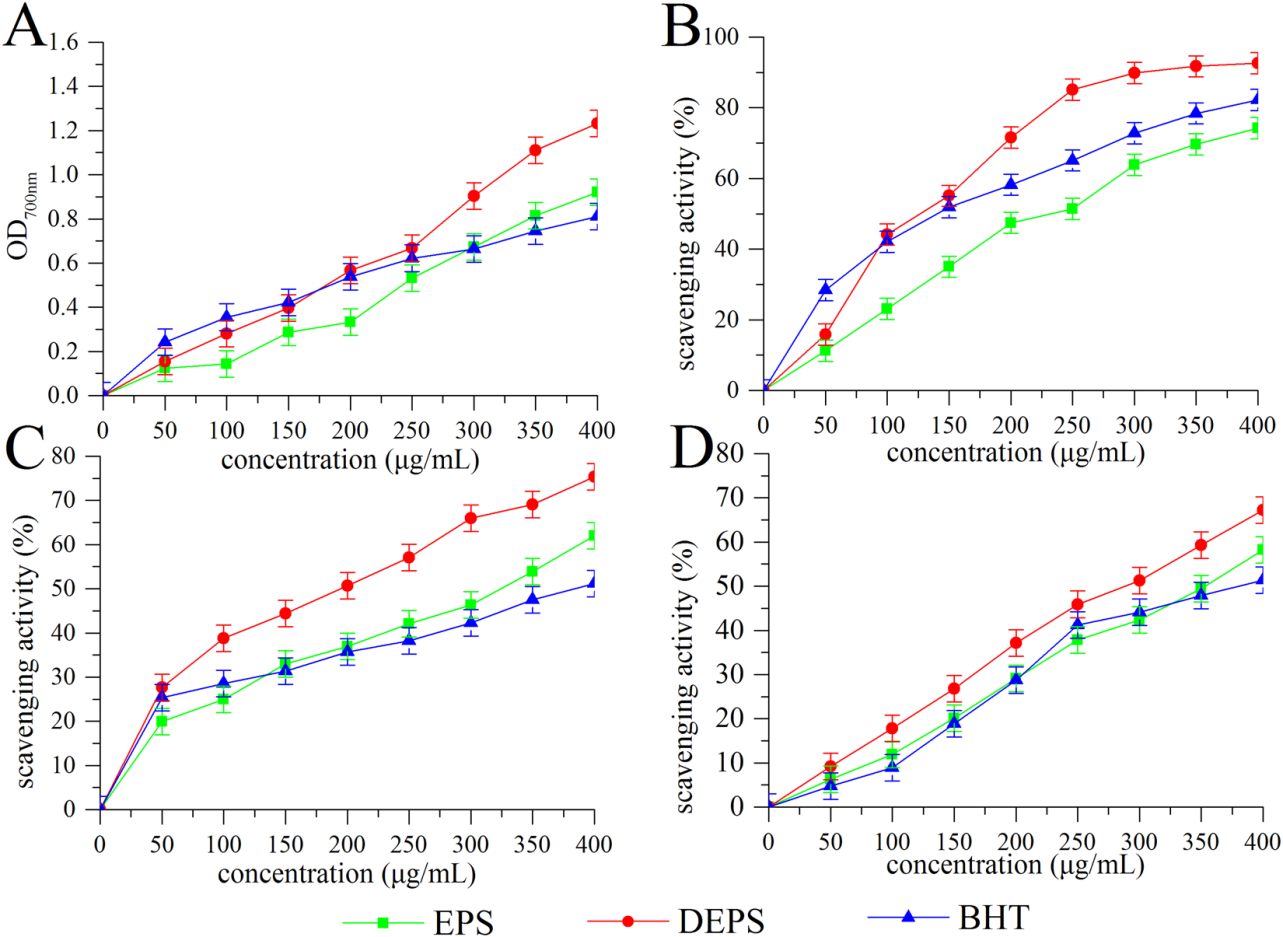
Antioxidant activities of EPS and DEPS *in vitro*. Reducing power (**A**) and scavenging activities towards DPPH radicals (**B**), hydroxyl radicals (**C**), and superoxide anion radicals (**D**).

The reducing power of EPS, DEPS and butylated hydroxytoluene (BHT) was shown in Fig. 1A. EPS, DEPS and BHT showed potential dose-dependent antioxidant activities. The reducing power of DEPS reached 1.233 ± 0.08, which was greater than the reducing power of EPS (0.921 ± 0.04) and BHT (0.811 ± 0.06) at a concentration of 400 μg/mL.

As shown in Fig. 1B, the scavenging activities of EPS, DEPS and BHT towards DPPH radicals significantly increased as the concentration increased. At a concentration of 400 μg/mL, the scavenging activity of DEPS (92.6 ± 5.12%) was stronger than the scavenging activity of EPS (74.27 ± 3.62%) or BHT (82.2 ± 4.72%). The IC_50_ values of EPS, DEPS and BHT were 214.47 ± 5.37, 118.28 ± 4.77 and 129.30 ± 4.86 μg/mL, respectively, indicating that DEPS showed a stronger scavenging activity than EPS and HBT.

As shown in Fig. 1C, EPS, DEPS and BHT showed obvious scavenging effects towards hydroxyl radicals, and the scavenging activity increased as the concentration increased. The hydroxyl radical scavenging activities of EPS, DEPS and BHT were 75.4 ± 4.71, 62.0 ± 3.18 and 51.2 ± 3.67%, respectively, at a concentration of 400 μg/mL. The IC_50_ values of EPS, DEPS and BHT were 312.90 ± 5.75, 162.11 ± 5.09 and 490.94 ± 6.26 μg/mL, respectively. The scavenging activity decreased in the order of DEPS, EPS, and BHT.

The scavenging activities of EPS, DEPS and BHT towards the superoxide radical increased as the concentration increased (Fig. 1D), and DEPS (67.21 ± 3.56%) exhibited a superior scavenging activity compared with EPS (58.20 ± 3.97%) and BHT (51.38 ± 2.97%). The IC_50_ values of DEPS reached 275.23 ± 5.62 μg/mL, which was lower than the IC_50_ of EPS (349.86 ± 5.86 μg/mL) and BHT (358.12 ± 5.88 μg/mL).

### Mouse weight analysis

As shown in Table 1, no significant differences in the initial body weight of the nine groups were observed. By the end of the experiment, the body weight of the mice in the high-fat emulsion group had increased significantly (*P* < 0.05) compared to the normal saline group. The body weights of mice in the dosage groups and the simvastatin group were significantly lower than the body weights of the mice in the high-fat emulsion group, indicating that both EPS and DEPS slowed the increase in body weight.

**Table 1:** Effects of EPS and DEPS on body weights.

Group	Body weight (g)	
	Initial body weights	Final body weights
Normal saline	19.82 ± 0.41	27.02 ± 0.59Eeε
High-fat emulsion	19.91 ± 0.39	29.01 ± 0.73Ddδ
Simvastatin	19.86 ± 0.40	27.37 ± 0.62Aaα
EPS		
100 mg/kg body weight	19.87 ± 0.45	28.73 ± 0.63B
200 mg/kg body weight	19.91 ± 0.39	28.36 ± 0.67b
400 mg/kg body weight	19.74 ± 0.31	27.67 ± 0.47β
DEPS		
100 mg/kg body weight	19.79 ± 0.37	28.18 ± 0.71C
200 mg/kg body weight	20.03 ± 0.47	27.82 ± 0.61c
400 mg/kg body weight	19.94 ± 0.42	27.39 ± 0.44γ

The values are reported as the means ± SD (n = 10). Bars with different letters are significantly different (*P* < 0.05).

### Serum lipid contents

**Figure 2:**
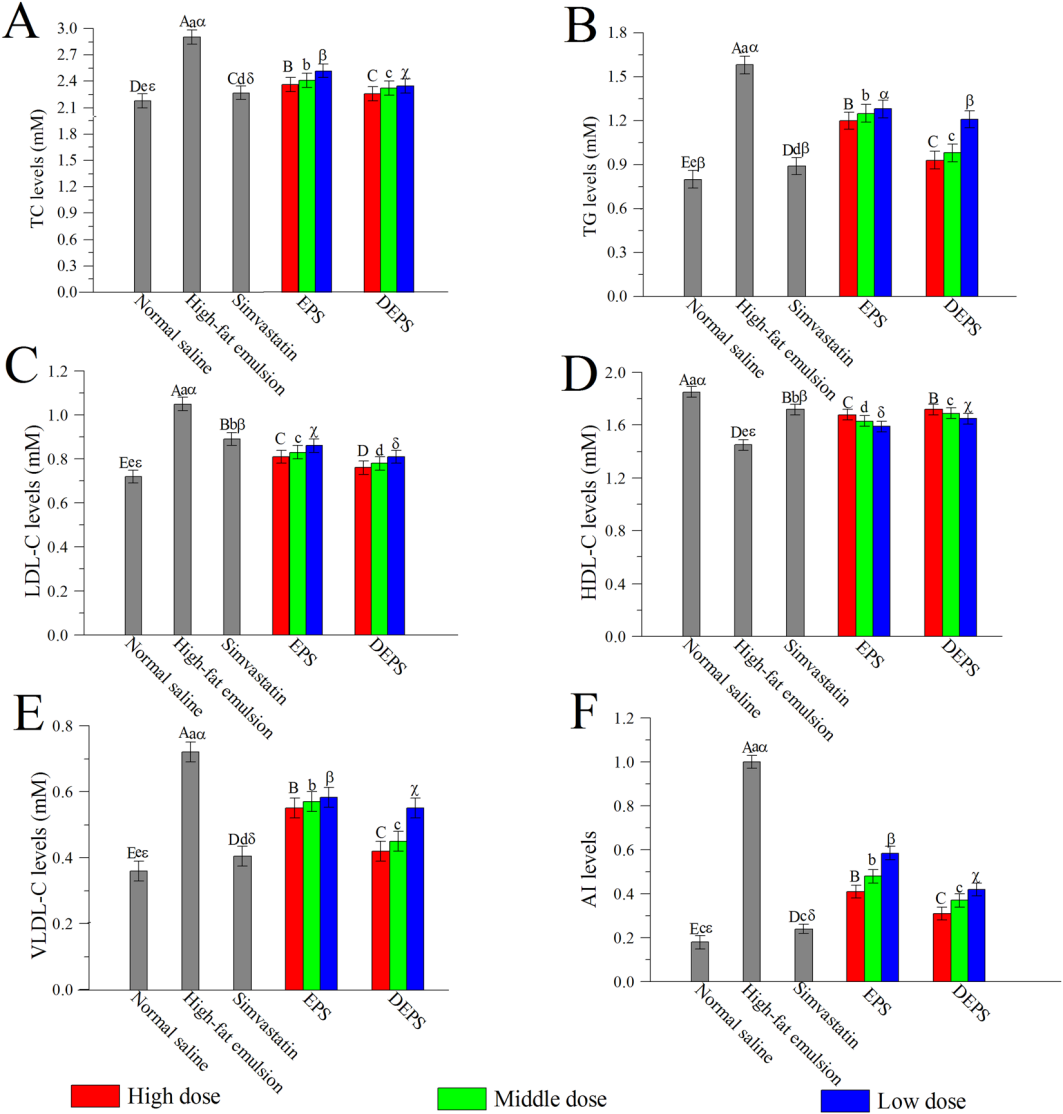
Effects of EPS and DEPS on serum lipid profiles in hyperlipidaemic mice: TC (**A**), TG (**B**), LDL-C (**C**), HDL-C (**D**), VLDL-C (**E**) and AI (**F**). The values are reported as means ± SD (n = 10). Bars with different letters are significantly different (*P* < 0.05).

The serum TC, TG, LDL-C, HDL-C and VLDL-C (very low density lipoprotein cholesterol) levels are presented in Fig. 2. Compared to the normal saline group, the high-fat emulsion significantly increased the TC, TG, LDL-C and VLDL-C levels and decreased the HDL-C levels in the high-fat emulsion group. Interestingly, the pathological changes were improved by the EPS and DEPS treatments, and DEPS exhibited superior effects compared to the other treatment groups (*P* < 0.05). In addition, the high AI (atherogenic index) was inhibited by the EPS and DEPS treatments, indicating that EPS and DEPS prevented and treated atherosclerosis (Fig. 2F).

### Hepatic lipid levels and liver index

**Figure 3:**
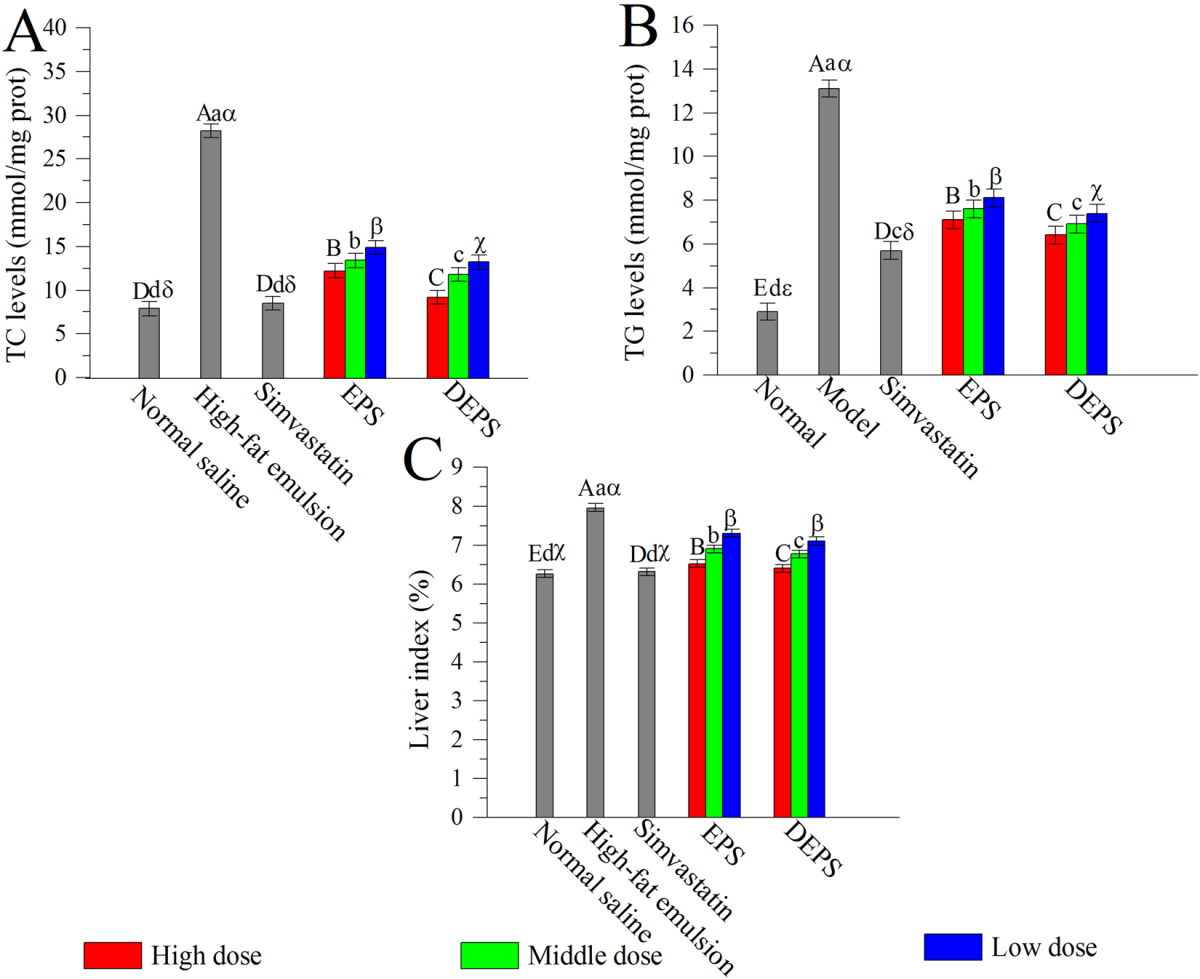
Effects of EPS and DEPS on liver lipid profiles in hyperlipidaemic mice: TC (**A**), TG (**B**) and liver index (**C**). Liver index (%) = liver weight/bodyweight × 100. The values are reported as means ± SD (n = 10). Bars with different letters are significantly different (*P* < 0.05).

As shown in Fig. 3A,B, the hepatic TC and TG levels in the high-fat emulsion group were significantly higher than the hepatic TC and TG levels in the normal saline and simvastatin groups (*P* < 0.05). Compared to the high-fat emulsion mice, the TC and TG levels in mice in the treated groups (EPS and DEPS) were significantly lower than the TC and TG levels in the high-fat emulsion mice (*P* < 0.05). The hepatic TC and TG levels in the high-dose EPS group decreased by 51.6% and 45.8%, and the TC and TG levels in the high-dose DEPS groups decreased by 67.4% and 51.1%, respectively. In addition, the liver index of mice was reduced by 19.1% and 19.9% in the high-dose EPS and DEPS groups, respectively (Fig. 3C). Based on these results, DEPS showed superior effects on lowering the serum lipid levels compared to EPS at a dose of 400 mg/kg body weight.

### Hepatoprotective activity

**Figure 4:**
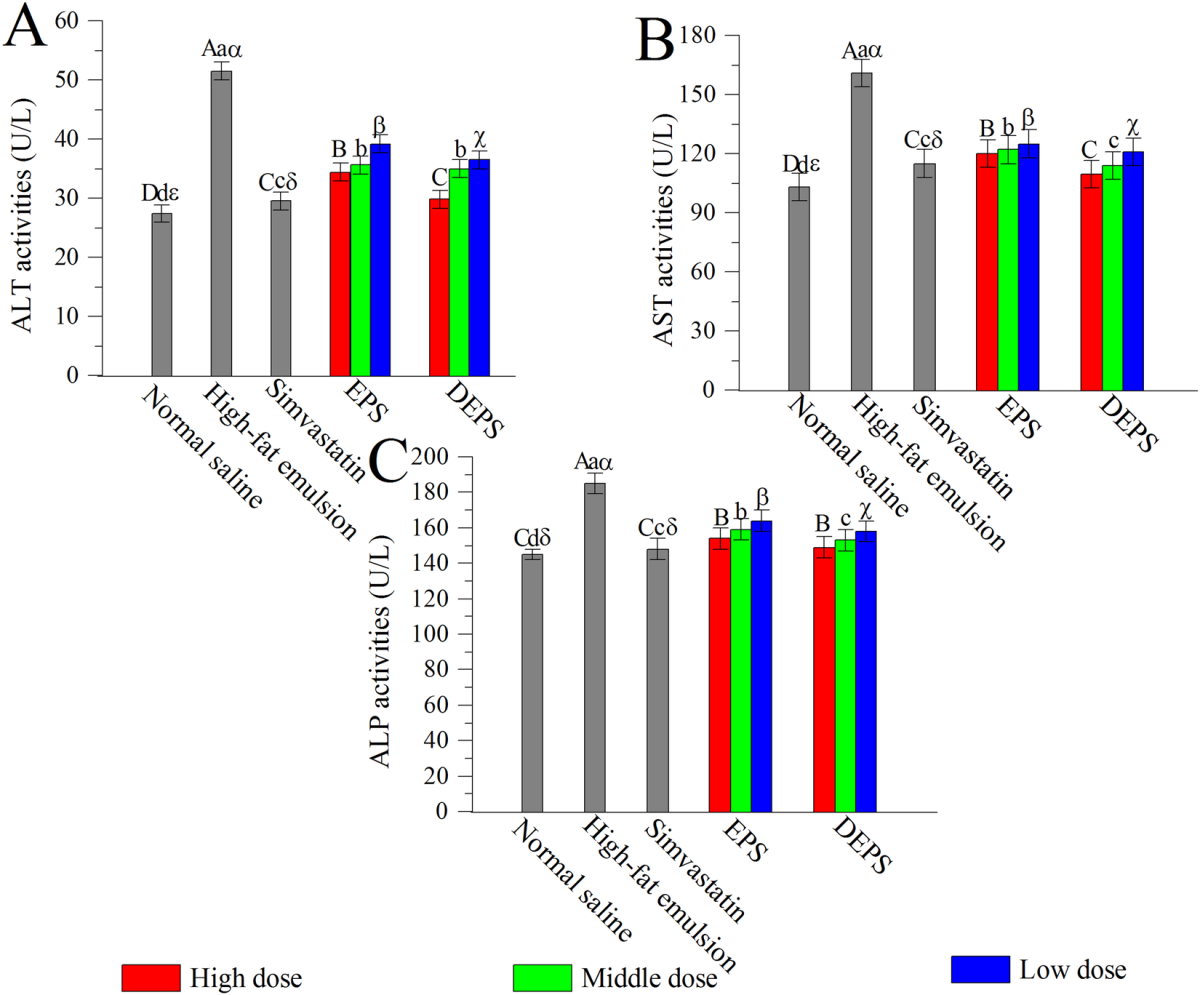
Effects of EPS and DEPS on the serum ALT (**A**), AST (**B**) and ALP (**C**) activities. The values are reported as means ± SD (n = 10). Bars with different letters are significantly different (*P* < 0.05).

The activities of several serum enzymes, including alanine aminotransferase (ALT), aspartate aminotransferase (AST) alkaline phosphatase (ALP), superoxide dismutase (SOD), GSH peroxide (GSH-Px), and catalase (CAT), as well as the total antioxidant capacity (T-AOC) and lipid peroxidation of malondialdehyde (MDA) and lipid peroxidation (LPO) in the liver were related to liver damage. The serum ALT, AST and ALP activities in the high-fat emulsion group were significantly higher than the serum activities observed in the normal saline group (*P* < 0.05, Fig. 4). However, these increases were controlled by treatments with the two polysaccharides (*P* < 0.05). Briefly, the ALT activity (29.8 ± 1.4 U/L), AST activity (109.5 ± 3.2 U/L) and ALP activity (149 ± 3.8 U/L) were reduced in the group treated with 400 mg DEPS/kg body weight compared with the group treated with the same dose of EPS.

**Figure 5:**
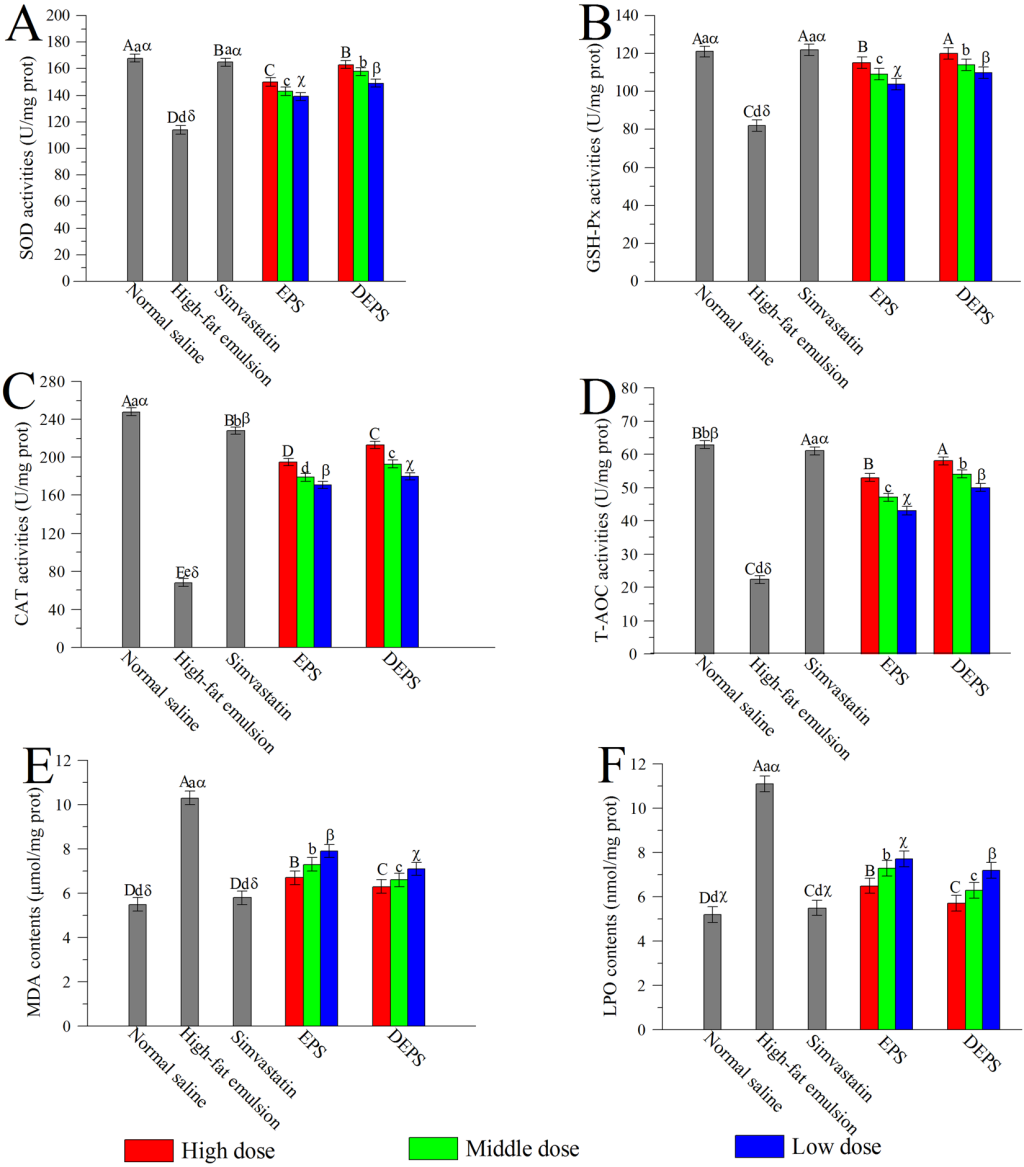
Effects of EPS and DEPS on SOD (**A**), GSH-Px (**B**), CAT (**C**), and T-AOC (**D**) activities and the MDA (**E**) and LPO (**F**) contents. The values are reported as means ± SD (n = 10). Bars with different letters are significantly different (*P* < 0.05).

Compared with the normal saline group, the hepatic SOD, GSH-Px, CAT and T-AOC activities in the high-fat emulsion groups were decreased, and the hepatic MDA and LPO contents were significantly increased (*P* < 0.05, Fig. 5A–F), indicating that severe oxidative stress occurred in the liver. After 25 days of lavage, the polysaccharide-treated groups exhibited significant increases in SOD, GSH-Px, CAT and T-AOC activities as well as remarkable decreases in the MDA and LPO contents compared with the high-fat emulsion group. The SOD, GSH-Px, CAT and T-AOC activities of the group treated with 400 mg DEPS/kg body weight reached maximum values of 163.1 ± 11.1, 120.4 ± 13.4, 213.3 ± 19.3 and 58.0 ± 4.2 U/mg prot, respectively, which were higher than the values observed in the group treated with the same dose of EPS (149.1 ± 14.1, 115 ± 12.2, 195 ± 15.2 and 53 ± 4.3 U/mg prot, respectively). The MDA and LPO content in the group treated with 400 mg DEPS/kg body weight reached 6.3 ± 0.55 μmol/mg prot and 5.7 ± 0.32 nmol/mg prot, respectively, which were lower than the values observed in the EPS group.

Simvastatin-treated (200 mg/kg body weight) mice also showed significant increases in SOD, GSH-Px, CAT and T-AOC activities as well as decreases in ALT, AST, and ALP activities and the MDA and LPO contents compared to the high-fat emulsion mice (*P* < 0.05).

### Histopathological observations

**Figure 6:**
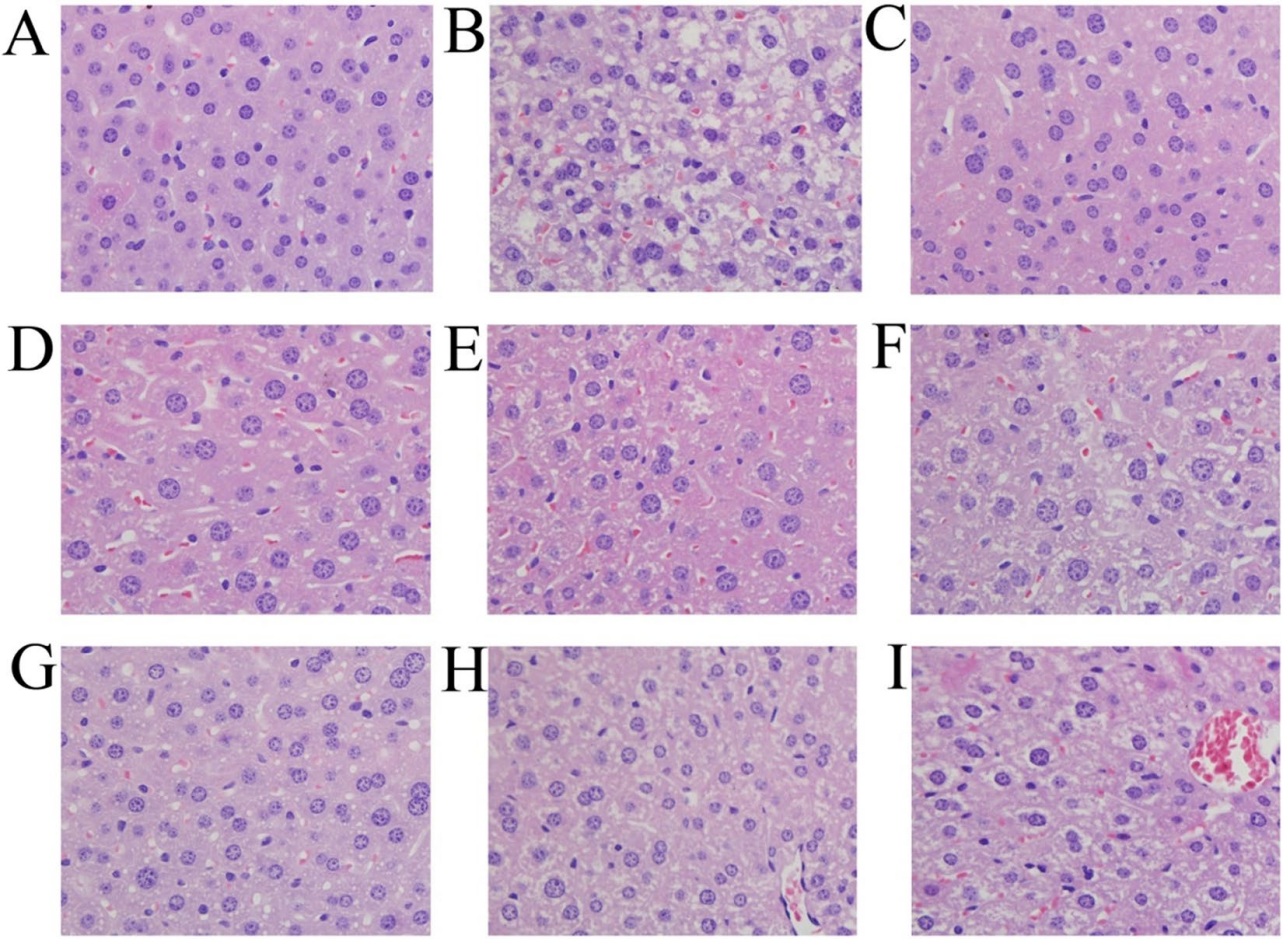
Optical micrographs of mouse liver sections (400 × magnification). Liver sections from mice in the normal saline group (**A**), high-fat emulsion group (**B**), simvastatin group (**C**), and groups treated with100, 200 and 400 mg/kg body weight EPS (**D**–**F**) of DEPS (**G**–**I**).

In the current study, light microscopic images of the hepatic histopathological findings are shown in Fig. 6. The liver cells in the normal saline group were arranged in an ordered manner and exhibited a normal cellular morphology, abundant cytoplasm, well-defined cell borders, no symptoms of fat degeneration and a distinct hepatic nucleus. However, liver cells with extreme swelling, around shape, hepatic steatosis and inflammatory changes were observed in the high-fat emulsion group. In addition, extensive fatty or vesicular degeneration was observed in the hepatocyte cytoplasm, and some nuclei had disappeared. Interestingly, after treatment with EPS and DEPS, fat vacuoles and hepatic degeneration were markedly decreased. In particular, the intervention with 400 mg DEPS/kg body weight induced a hepatocyte morphology and arrangement that were similar to the control group. Thus, both EPS and DEPS derived from *T*. *albuminosus* obviously inhibited the high-fat emulsion-induced morphological changes and steatosis of liver cells.

### Acute toxicity study

None of the mice displayed abnormal behaviours after treatment with EPS and DEPS, even at a dose of 2000 mg/kg body weight. In addition, no deaths occurred throughout the experiment. Thus, EPS and DEPS were practically non-toxic substances.

### Preliminary characterization of EPS and DEPS

**Figure 7:**
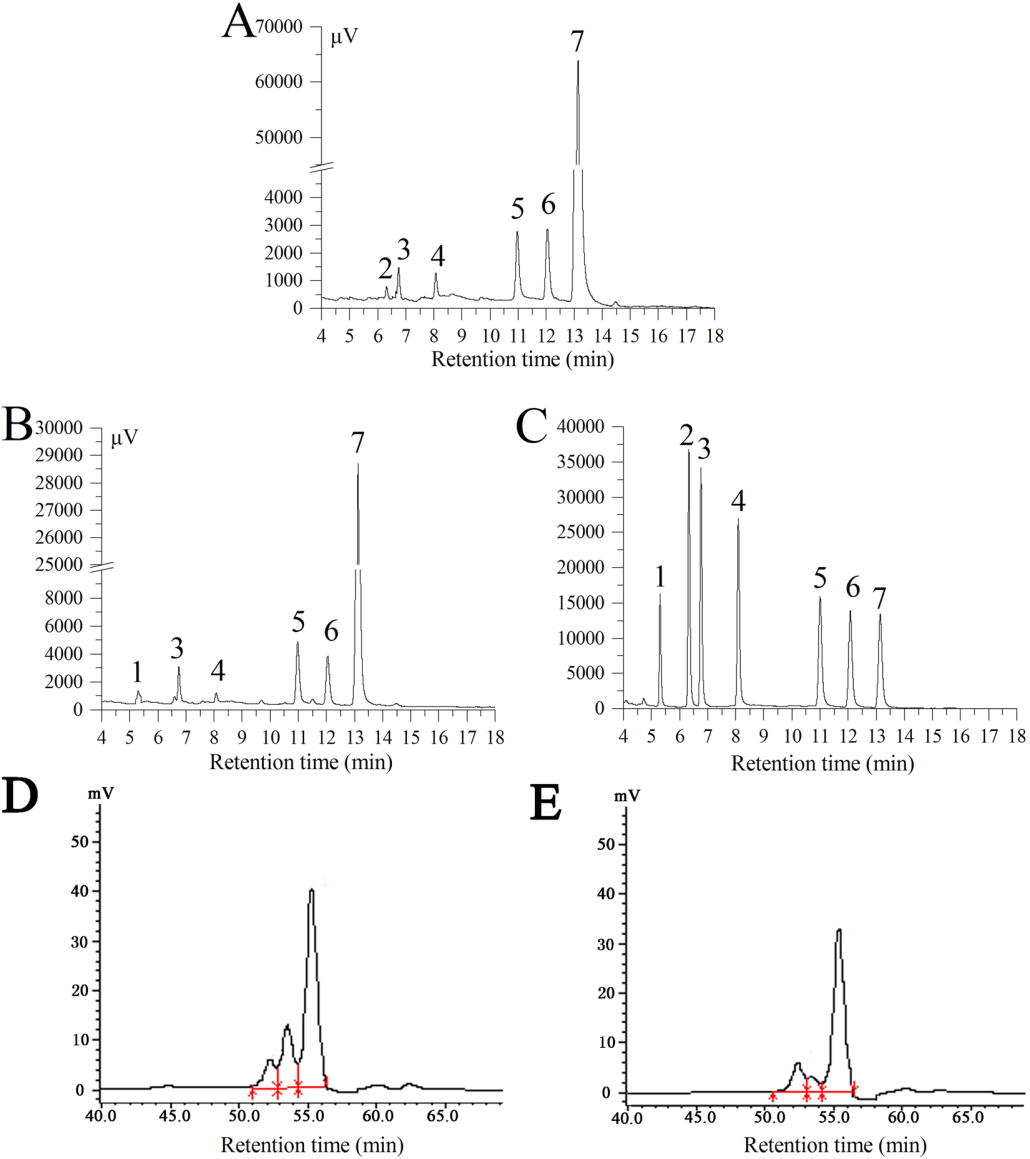
GC chromatograms of EPS (**A**), DEPS (**B**) and standard monosaccharides (**C**) as well as HPLC chromatograms of EPS (**D**) and DEPS (**E**) are shown. Peaks: (1) rhamnose, (2) ribose, (3) arabinose, (4) xylose, (5) mannose, (6) galactose, and (7) glucose.

The monosaccharide composition of EPS and DEPS was analysed based on a comparison of the retention time of the chromatographic peaks and monosaccharide reference samples (Fig. 7A). EPS consisted of six different monosaccharides, ribose, arabinose, xylose, mannose, galactose and glucose, with mass percentages of 3.16, 6.94, 2.34, 18.76, 20.2 and 48.6%, respectively (Fig. 7B). DEPS was composed of six monosaccharides, rhamnose, arabinose, xylose, mannose, galactose and glucose, with mass percentages of 4.24, 13.88, 1.56, 23.29, 18.63 and 38.4% (Fig. 7C), respectively.

Based on the results of the HPLC analysis, the Mw (weight-average molecular weight), Mn (number-average molecular weight) and Mz (z-average molecular weight) of EPS were 3.03 × 10^3^, 2.01 × 10^3^, and 2.52 × 10^3^ Da, whereas the Mw, Mn and Mz of DEPS were 1.43 × 10^3^, 1.22 × 10^3^, 1.46 × 10^3^ Da, respectively (Fig. 7D and E).

## Discussion

Currently, the use of polysaccharides from edible mushrooms as natural medicines has garnered increasing attention due to their important roles in the treatment of many pathological diseases and lack of negative consequences compared with synthetic drugs. Many academic researchers have focussed their attention the study of natural polysaccharides. An accumulating number of studies have presented many methods for modifying polysaccharides, including sulphation, acetylation, carboxymethylation, benzoylation, methylation, hydroxyethylation, hydroxypropylation, acid hydrolysis, enzymatic hydrolysis and alkaline hydrolysis^17–23^. In addition, many previous studies have proven that depolymerised-polysaccharides processed by enzymes possess superior physicochemical properties, such as good water solubility and high stability; moreover, they are safe and non-toxic and have higher biological activity than unmodified polysaccharides both *in vitro* and *in viv*o^17, 23^. However, the hepatoprotective effects of EPS and its modified form, DEPS, from *T*. *albuminosus* have not yet been studied.

Oxidative stress, a major contributor to many human diseases, including cardiovascular disease, atherosclerosis, inflammation, cancer, drug toxicity, reperfusion injury, neurodegeneration and liver diseases, is induced by ROS overproduction and an imbalance in the antioxidant capacity of the cell^24^. Antioxidant activities assessed using many different reactions, including radical scavenging activity, reductive capacity, binding to metalion catalysts to induce a transition, and the prevention of the chain initiation reaction^25^. Antioxidants have consistently been shown to decrease oxidative stress and slow/stop the development of disease complications by counteracting ROS production^26^. In the present work, the *in vitro* antioxidant activities of EPS and DEPS derived from *T*. *albuminosus* were investigated using four different parameters: reducing power and scavenging activity towards DPPH as well as hydroxy and superoxide anion radicals. According to previous reports, the reducing power, prevention of the chain initiation reaction and radical scavenging activity indicate positive antioxidant effects via the interruption of the free radical chain and the simultaneous contribution of a hydrogen atom to Fe^3+^, generating Fe^2+^ ^27–29^. The scavenging activity towards the DPPH radical, a jarless free radical, is a quick and effective indicator that reflects antioxidant activity when DPPH interacts with electron/hydrogen radicals to form non-radical form (DPPH-H)^30^. As a major type of ROS, the hydroxyl radical damages biomolecules and induces lipid peroxidation, which is not completely eliminated by the human defence system alone^24, 31^. Meanwhile, the scavenging capacity of polysaccharides towards hydroxyl radicals is directly correlated with their antioxidant activity^32^. Additionally, the superoxide anion radical, a relatively weak radical compared to other free radicals and oxidizing agents, is a precursor of singlet oxygen and hydroxyl radicals that activate lipid peroxidation and are generated by the xanthine/xanthine oxidase system^33^. In the present study, EPS and DEPS displayed remarkable reducing power and scavenging capacity towards the DPPH, hydroxyl and superoxide anion radicals, indicating that both EPS and DEPS possessed potential antioxidant activity. Compared with other reports, DEPS also showed an antioxidant capacity superior to the same concentrations of EIPS (enzymatic intracellular polysaccharides), IPS (intracellular polysaccharides) and EPS from *Flammulina velutipes* ^15, 23^. Based on these results, DEPS extracted from *T*. *albuminosus* shows potential for development as a natural antioxidant that prevents and treats diseases induced by ROS.

A high-fat emulsion has been widely used to induce hyperlipidaemia in mice^17^. Based on published pathological and epidemiological data, the hyperlipidaemia plays a pivotal role in accelerating the development of atherosclerosis and cardiovascular diseases^34^. The aim of this work was to examine the antihyperlipidaemic effects of EPS and DEPS on mice with high-fat emulsion-induced hyperlipidaemia. High serum HDL-C levels are regarded as “beneficial” to human health because excess HDL-C transport free cholesterol/cholesterol esters from peripheral tissues/cells through the blood circulation to the liver for catabolism by the “reverse cholesterol transport” pathway^35^. However, in the presence of high serum LDL-C (the main carrier of cholesterol) levels, LDL-C may be transported to the endarterium, where oxidized LDL-C (ox-LDL-C) is generated, potentially decreasing the interactions between LDL-C and specific membrane receptors and increasing its permanence in the bloodstream^36^. These structural and functional changes in LDL may accelerate the development of atherosclerotic plaques in blood vessels, resulting in increased levels of lipid peroxidation products (MDA)^17^. Furthermore, high serum TC and TG levels may increase the blood viscosity, potentially inducing the development of hyperlipidaemia and atherosclerosis. Therefore, drugs that decrease serum TC, TG, LDL-C and VLDL-C levels and increase serum HDL-C levels seem to be required to treat hyperlipidaemia and atherosclerosis. In this work, blood lipid levels were significantly improved in the high-fat emulsion-induced hyperlipidaemic mice following the gavage of EPS and DEPS, indicating that both EPS and DEPS displayed potential therapeutic effects on hyperlipidaemia.

Serum AST, ALT and ALP activities have been used as biochemical criteria to evaluate liver function, and the activities of these enzymes increase significantly upon liver damage^37^. The leakage of enzymes from hepatocytes into the bloodstream is associated with the increased permeability of the cell membrane^38^. The hepatic TC and TG contents could also reflect the accumulation of fat, and the presence of a lipochondrion on the surface of the hepatocyte may decrease liver function. In this study, the serum AST, ALT and ALP activities and hepatic TC and TG contents were significantly decreased by the EPS and DEPS treatments compared to the high-fat emulsion group, indicating that both EPS and DEPS potentially treated liver damage caused by hyperlipidaemia. DEPS exhibited superior effects compared to EPS. Furthermore, the results of the liver slice analysis were consistent with biochemical indicators of liver function.

In addition, the hepatic activities of antioxidant enzymes (SOD, GSH-Px and CAT) and lipid contents (MDA and LPO) were determined to investigate the antioxidant effects of EPS and DEPS on liver damage in the high-fat emulsion-induced hyperlipidaemic mice. Antioxidant enzymes, the first line of defence against oxidative damage in mammalian systems, catalyse the degradation of ROS into innoxious compounds, defending against ROS-induced oxidative stress in the organism^39^. SOD, the most important antioxidant enzyme that defends against oxidative stress, converts superoxides to hydrogen peroxide^8^. Hydrogen peroxide is subsequently degraded into less-reactive gaseous oxygen and water by CAT^17^. In addition, as a selenium-containing enzyme, GSH-Px specifically catalyses the reduction of GSH to H_2_O_2_to protect the integrity and function of the plasma membrane^6^. The capacity of the non-enzymatic antioxidant defence system in the organs was determined by analysing T-AOC activity. In addition, MDA, one of the toxic aldehyde compounds created by lipid peroxidation, is considered the most important oxide derivative generated during lipid metabolism, and LPO is the product of reaction between oxygen radicals and polyunsaturated fatty acids^40, 41^. Hence, the formation of MDA and LPO in the liver is thought to be the main cause of liver damage^42^. In this study, the mice in the high-fat emulsion group displayed significant decreases in SOD, GSH-Px, CAT and T-AOC activities and increased MDA and LPO contents compared to the mice in the normal saline group, indicating that serious oxidative injury had occurred in the liver. However, noteworthy reductions in the MDA and LPO contents and increases in the SOD, CAT, GSH-Px and TAOC activities were observed after the administration of polysaccharides (EPS and DEPS). Based on these experimental results, both EPS and DEPS obviously reduced liver damage caused by high-fat emulsion-induced oxidative stress. However, the clinical mechanisms by which the two polysaccharides treat hyperlipidaemia and subsequent liver damage should be explored in future studies.

The antioxidant activities of polysaccharides are mainly associated with their characteristics, such as their monosaccharide compositions and molecular weights^43^. The monosaccharide compositions and molecular weights of EPS and DEPS were investigated in this study. The major components of EPS and DEPS were mannose, galactose and glucose, which were different from the monosaccharide compositions of other EPS from *F*. *velutipes* ^15^ and *Hericium erinaceus* ^16^, possibly due to differences in the culture medium, strains, fermentation conditions and extraction conditions. Furthermore, rhamnose was detected only in DEPS, indicating that rhamnose may have important contributions to the antioxidant activities of polysaccharides. Similar compositions were observed mycelium zinc polysaccharides (MZPS) from *Pleurotus djamor* and enzymatic mycelium polysaccharides from *T*. *albuminosus* (EIPS) and *F*. *velutipes* (EIPS)^9, 17, 23^. According to the results of molecular weight assays, low molecular weight components may be responsible for the increased antioxidant activity of DEPS, consistent with the results of previous studies of polysaccharides extracted from *Ganoderma lucidum* (GLPL1)^44^ and *Tricholoma lobayense* (TLH-3)^45^.

## Materials and Methods

### Chemicals

The DEAE-52 cellulose anion-exchange resin was purchased from Whatman Chemical Company (UK). DPPH was purchased from Sigma Chemical Company (St. Louis, USA). The diagnostic kits used to analyse SOD activity, GSH-Px activity, CAT activity, T-AOC activity, LPO contents, and MDA contents were purchased from Nanjing Jiancheng Bioengineering Institute (Nanjing, China). The standard monosaccharide samples were provided by Merck Company (Darmstadt, Germany) and Sigma Chemical Company (St. Louis, USA). All other chemicals used in the present work were analytical reagent grade and supplied by local chemical suppliers.

### Strain and culture

The strain of *T*. *albuminosus* used in the present work was provided by our laboratory and maintained on potato dextrose agar (PDA) slant. The *T*. *albuminosus* fermentation broth was obtained using liquid fermentation technology. The seed culture in liquid media was processed in a 1-L filter flask containing 500 mL of 200 g/L potato, 20 g/L glucose, 1.5 g/L KH_2_PO_4_, and 1 g/L MgSO_4_·7H_2_O. Subsequently, the seeding broth was inoculated into the fermentation tank (100-L, Xianmin, China) and cultivated for 10 days (25 °C, natural pH).

### Preparation of EPS and DEPS

The *T*. *albuminosus* fermentation broth was isolated by filtration and concentrated with an Electro-Thermostatic Blast Oven (DHG-9143B5-III, Shanghai, China) at 60 °C. The concentrated solution was precipitated with three volumes of ethanol (95%, v/v) and incubated at 4 °C overnight. The resulting precipitate was separated by centrifugation (3,000 r/min, 10 min), the proteins were removed using the Sevag method and dialysing against distilled water to yield EPS^17^.

DEPS was prepared using the method reported by Yang, Gao, and Han^46^. The EPS powder (1 g) and snailase (0.2 g) were dissolved in 50 mL of sodium acetate buffer (1%, w/v). The enzymatic mixture was processed under the following conditions: a pH ranging from 4 to 5.2 at 37 °C, for 3 h. Both EPS and DEPS were washed with ultrapure water and then lyophilized for further analysis.

### Total polysaccharide analysis

The polysaccharide content was determined by measuring the absorbance at 490 nm using the phenol-sulphuric acid colorimetric method and glucose as the standard^47^. All crude polysaccharides were obtained from a 100-L fermentation tank containing 80 L of fermentation broth. The dried crude polysaccharide powder (2 g) was dissolved in 10 mL of deionized water and purified. The purified solution was collected, precipitated, lyophilized and weighed.

### Analysis of the antioxidant effects *invitro*

#### Reducing power assay

The reducing power of EPS and DEPS was measured using the methods reported by Oyaizu^48^. The tested sample (1 mL, 0–400 μg/mL), phosphate buffer (2.5 mL, 0.2 mol/L, pH 6.6) and potassium ferricyanide (1.0 mL, 1%, w/v) were mixed and incubated at 50 °C for 20 min. Subsequently, trichloroacetic acid (2 mL, 10%, w/v) and ferric chloride (1.2 mL, 0.1%, w/v) were added to the reaction mixture to terminate the reaction. The absorbance was measured at 700 nm using deionized water as a blank and BHT as a positive control.

#### Scavenging activity towards DPPH radicals

The scavenging activities of EPS and DEPS towards DPPH radicals were measured using a previously reported method^49^. The sample (2 mL, 0–400 μg/mL), DPPH (2 mL, 2 × 10^−4^ mol/L) and anhydrous ethylalcohol (2 mL) were mixed uniformly. The mixture was incubated for 30 min in the dark, and the absorbance was recorded at 517 nm using absolute ethanol as the blank and BHT as a positive control for a comparison. The IC_50_ value (μg/mL) of samples or BHT is the effective concentration at which 50% of the DPPH radicals were scavenged. The original colour of the liquid becomes lighter or even colourless, reflecting the increasing scavenging activity toward DPPH radicals, and the scavenging rate is expressed as:

1 $${\rm{Scavenging}}\,{\rm{rate}}\,( \% )=[1-({{\rm{A}}}_{{\rm{i}}}-{{\rm{A}}}_{{\rm{j}}}){/{\rm{A}}}_{{\rm{c}}}]\times 100$$

where A_c_ is the absorbance of DPPH (2 mL) and anhydrous ethylalcohol (2 mL), A_i_ is the absorbance of the sample (2 mL) and DPPH (2 mL) and A_j_ is the absorbance of sample (2 mL) and anhydrous ethylalcohol (2 mL), respectively.

#### Scavenging activity towards hydroxyl radicals

The hydroxyl radical scavenging ability was measured using the method reported by Winterbourn and Sutton^50^. The reaction mixtures contained ferrous sulphate (1 mL, 9 mmol/L), salicylic acid (1 mL, 9 mmol/L), samples (1 mL, 0–400 μg/mL) and hydrogen peroxide (1 mL, 8.8 mmol/L) and were incubated at 37 °C for 30 min. After centrifugation (1,200 r/min for 6 min), the absorbance was measured at 510 nm using deionized water instead as a blank and BHT as a positive control. The scavenging rate was calculated using the following equation:

2 $${\rm{Scavenging}}\,{\rm{rate}}\,( \% )=[({{\rm{A}}}_{{\rm{0}}}-{{\rm{A}}}_{{\rm{1}}}){/{\rm{A}}}_{{\rm{0}}}]\times 100$$

where A_0_ is the absorbance of the blank (deionized water instead of sample and reagents) and A_1_ is the absorbance of the sample.

#### Scavenging activity towards superoxide anion radicals

The scavenging activity towards superoxide anion was determined using a previously reported method^9^. The reaction mixtures contained Tris-HCl buffer (4.5 mL, pH 8.2, 50 mmol/L), deionized water (3.2 mL) and samples (1 mL, 0–400 μg/mL) and were incubated at 25 °C for 20 min. Pyrogallol (0.3 mL) was added and the mixture was incubated at 25 °C for an additional 20 min. After adding vitamin C (1 mL, 5% w/v) to terminate the reaction, the absorbance was measured at 420 nm using deionized water as a blank and BHT as a positive control. The scavenging rate was evaluated using the following formula:

3 $${\rm{Scavenging}}\,{\rm{rate}}\,( \% )=[({{\rm{A}}}_{{\rm{0}}}-{{\rm{A}}}_{{\rm{1}}}){/{\rm{A}}}_{{\rm{0}}}]\times 100$$

where A_0_ is the absorbance of the blank (deionized water instead of sample and reagents), and A_1_ is the absorbance of samples.

#### Animal experiments

The high-fat emulsion was prepared using the method described by Wang *et al*.^35^. The oil phase consisted of a mixture of 25 g of lard oil, 10 g of cholesterol, 1 g of methylthiouracil and 25 mL of Tween-80. The water phase consisted of 30 mL of distilled water, 20 mL of propylene glycol and 2 g of sodium deoxycholate. The oil phase and water phase were mixed to obtain the high-fat emulsion.

The ninety male Kunming mice (20 ± 2 g) used in the animal experiments were purchased from Taibang Biological Products Co., Ltd. (Taian, China) and housed in a standard room (temperature 22 ± 1 °C, relative humidity 50 ± 5%, and a 12-h light/dark cycle). The experiments were performed according to procedures approved by the Institutional Animal Care and Use Committee of Shandong Agricultural University in accordance with the Animals (Scientific Procedures) Act. 1986 (amended 2013). All animals had access to food and water *ad libitum*.

After a week of adaptive feeding, all mice were randomly divided into nine groups (10 mice in each group) including one normal saline group, one high-fat emulsion group, one simvastatin group, three EPS-treated groups (100, 200 and 400 mg/kg body weight) and three DEPS-treated groups (100, 200 and 400 mg/kg body weight). During the experimental procedure, the EPS- and DEPS-treated groups received alternating daily gavages of the high-fat emulsion and polysaccharides, whereas the normal saline groups received normal saline, the high-fat emulsion groups received high-fat emulsion, and the simvastatin groups received simvastatin (200 mg/kg body weight) as a control. The entire study lasted for 25 consecutive days.

At the end of the experiment, all mice were weighed, fasted for 12 h, and euthanized. Blood samples were collected from the retrobulbar vein and the serum was obtained by centrifugation (15,000 r/min for 15 min). The livers were quickly excised, weighed, and homogenized (1:9, w/v) with normal saline and ethyl alcohol. After centrifugation (5,000 r/min for 10 min), the supernatant was collected and stored at −4 °C until further analysis.

The serum ALT, ALP and AST activities and TC, TG, HDL-C, LDL-C, and VLDL-C levels were determined using an automatic biochemical analyser (BS-380, Shenzhen, China). The AI was calculated as (TC - HDL-C)/HDL-C^12^.

The hepatic SOD, GSH-Px, CAT and T-AOC activities, MDA and LPO contents, and TC and TG levels were analysed using commercial kits according to the manufacturer’s instructions.

Fresh liver tissues were fixed with a 4% formaldehyde solution, embedded in paraffin, cut into slices, and stained with haematoxylin and eosin. Each section was observed under a microscope (×400 magnification).

#### Acute toxicity study

Eighteen male Kunming mice (20 ± 2 g) were randomly divided into three groups (six mice each group) and used for the acute toxicity study, as previously described^51^. The mice in the experimental group were administered EPS and DEPS at increasing doses of 500, 1000, 1500 and 2000 mg/kg body weight, respectively; the normal saline mice administered the same volume of normal saline. During the experiment, all mice were provided with food and water *ad libitum*, and observed for mortality and behavioural changes for 48 h.

#### Preliminary characterization of EPS and DEPS

The monosaccharide composition was determined using a GC (GC-2010, Shimadzu, Japan) equipped with a hydrogen flame ionization detector (FID) based on our previously reported method^17^. The samples were sequentially hydrolysed, acetylated and analysed. Monosaccharide components were analysed by comparing the samples with standard monosaccharides: arabinose, xylose, ribose, galactose, mannose, rhamnose, and glucose.

Molecular weights and homogeneities were determined using an HPLC system (Shimadzu LC-2010AT, Japan) equipped with an Atlantis C18 column (250 mm × 4.6 mm × 5 µm) and a refractive index detector. The sample (20 µL) was injected into the Atlantis C18 column using deionized water as the mobile phase at a flow rate of 1 mL/min, and the column temperature was maintained at 30 °C. A series of standard dextran solutions was used to generate the calibration curve. The molecular weights were analysed using Agilent GPC software.

#### Statistical analysis

SAS was used for the statistical evaluation. Data are expressed as means ± SD (standard deviations). Statistical analyses were performed using one-way ANOVA. Significant differences between experimental groups were determined using Tukey’s tests, and *P* < 0.05 was considered a statistically significant difference.

## Conclusions

In conclusion, EPS and DEPS from *T*. *albuminosus* exerted pharmacological *in vitro* antioxidant, hepatoprotective and hypolipidaemic effects on mice with high-fat emulsion-induced hyperlipidaemia. Both EPS and DEPS are potential natural sources that may prevent hyperlipidaemia and oxidative injury induced by high-fat emulsions. In addition, specific components of DEPS, including low molecular weight compounds and rhamnose, potentially increased the bioactivity of this polysaccharide.
